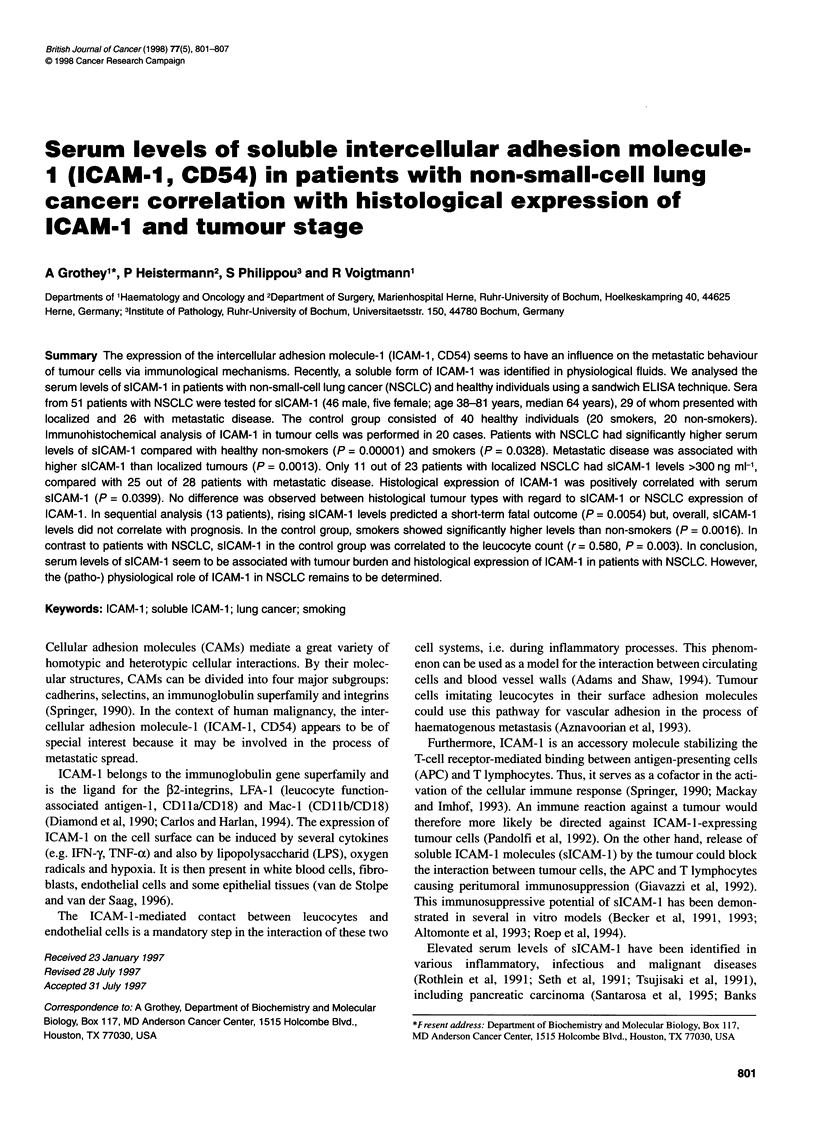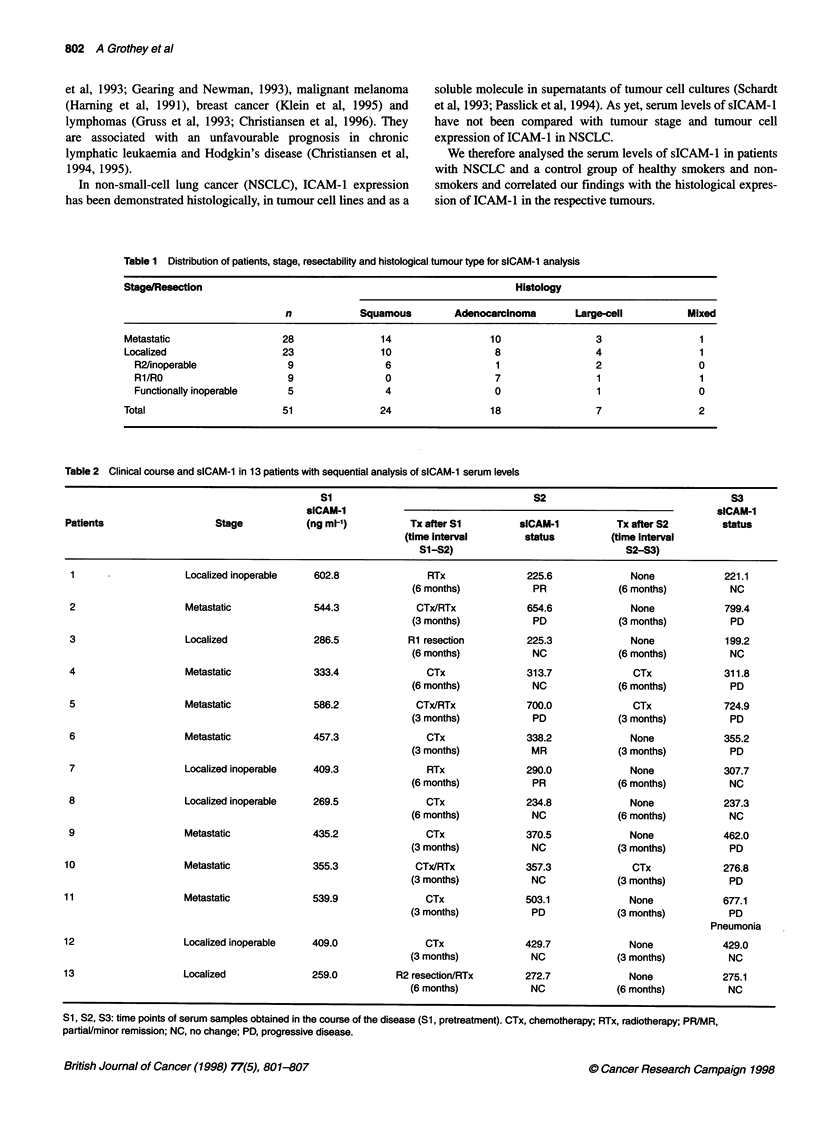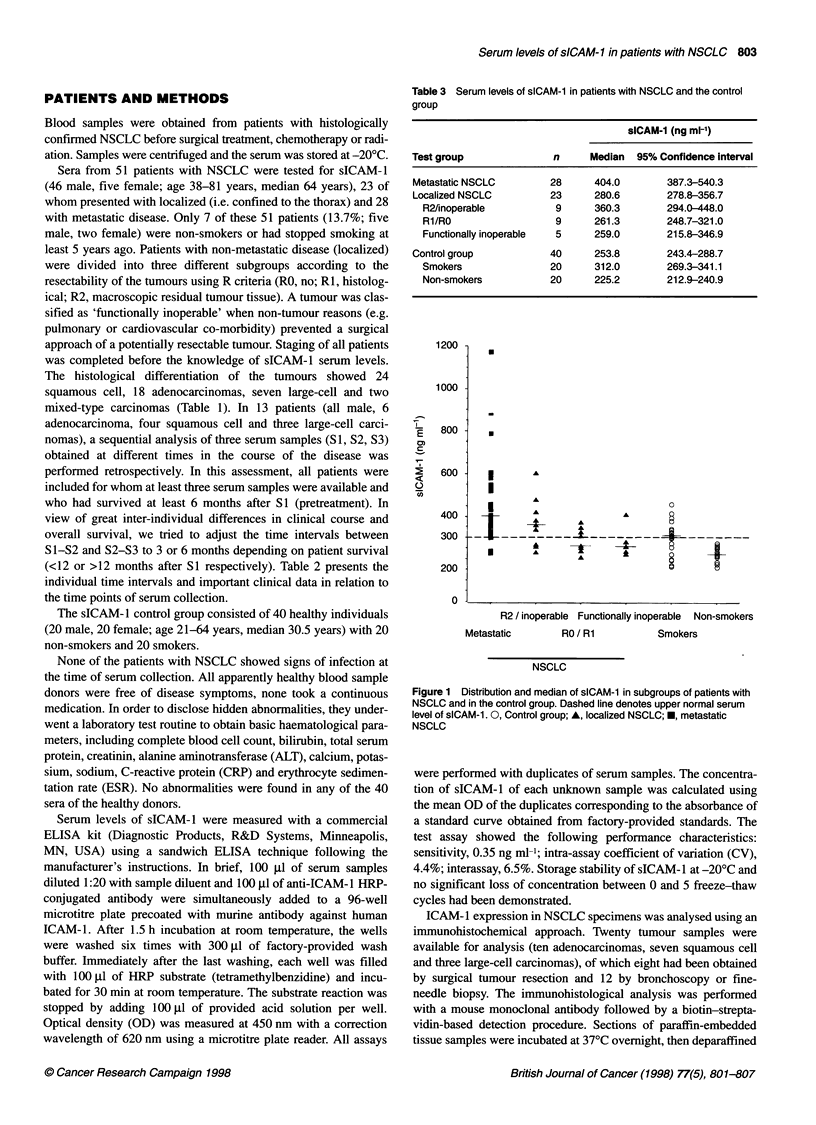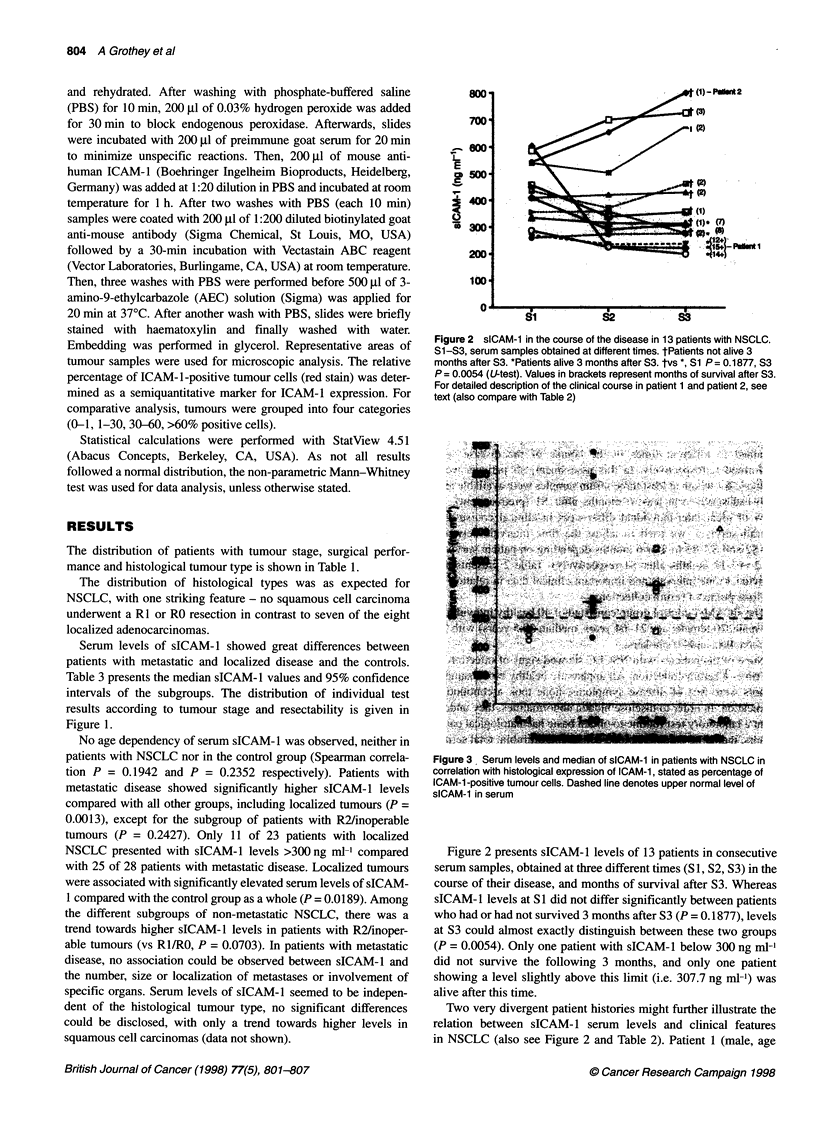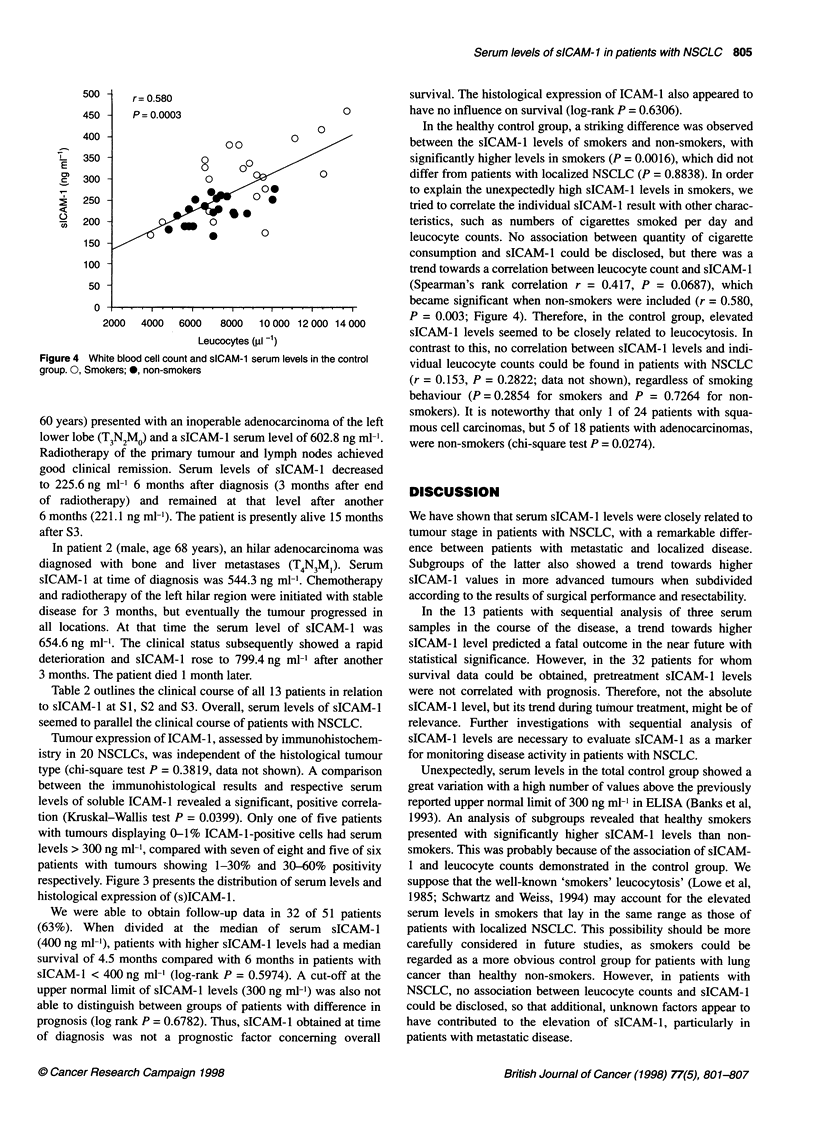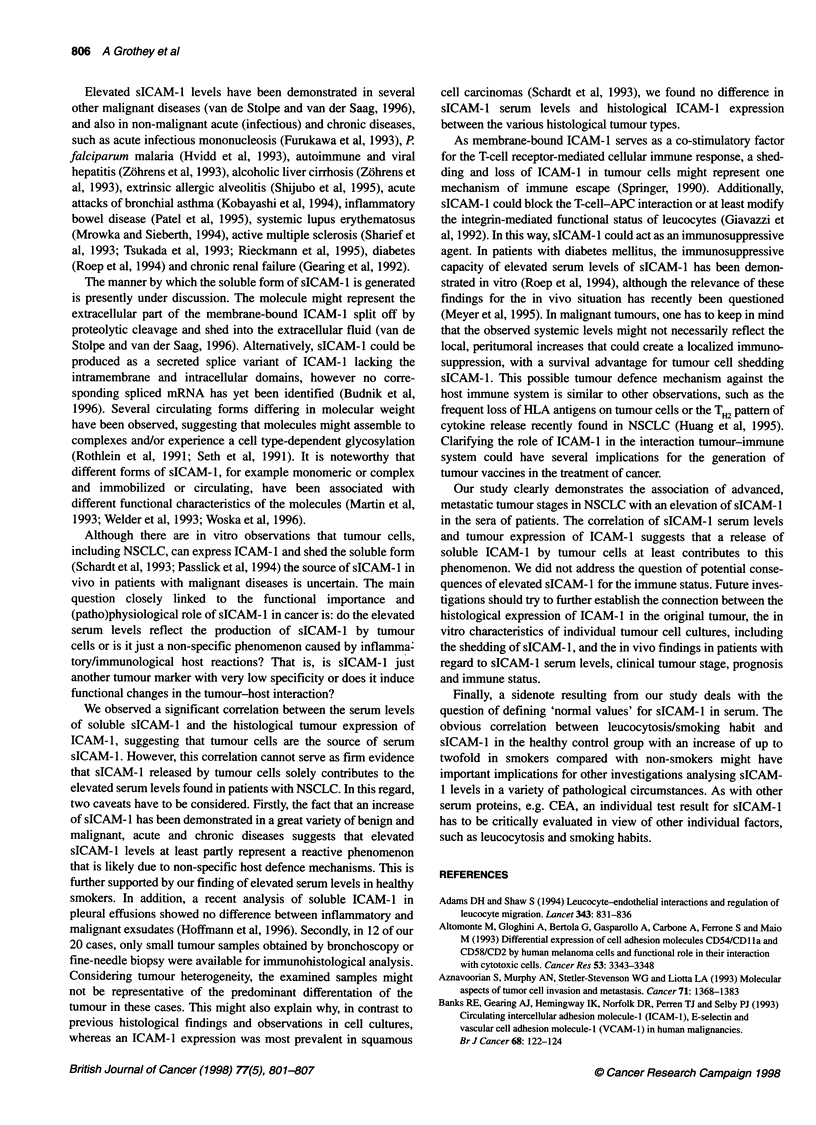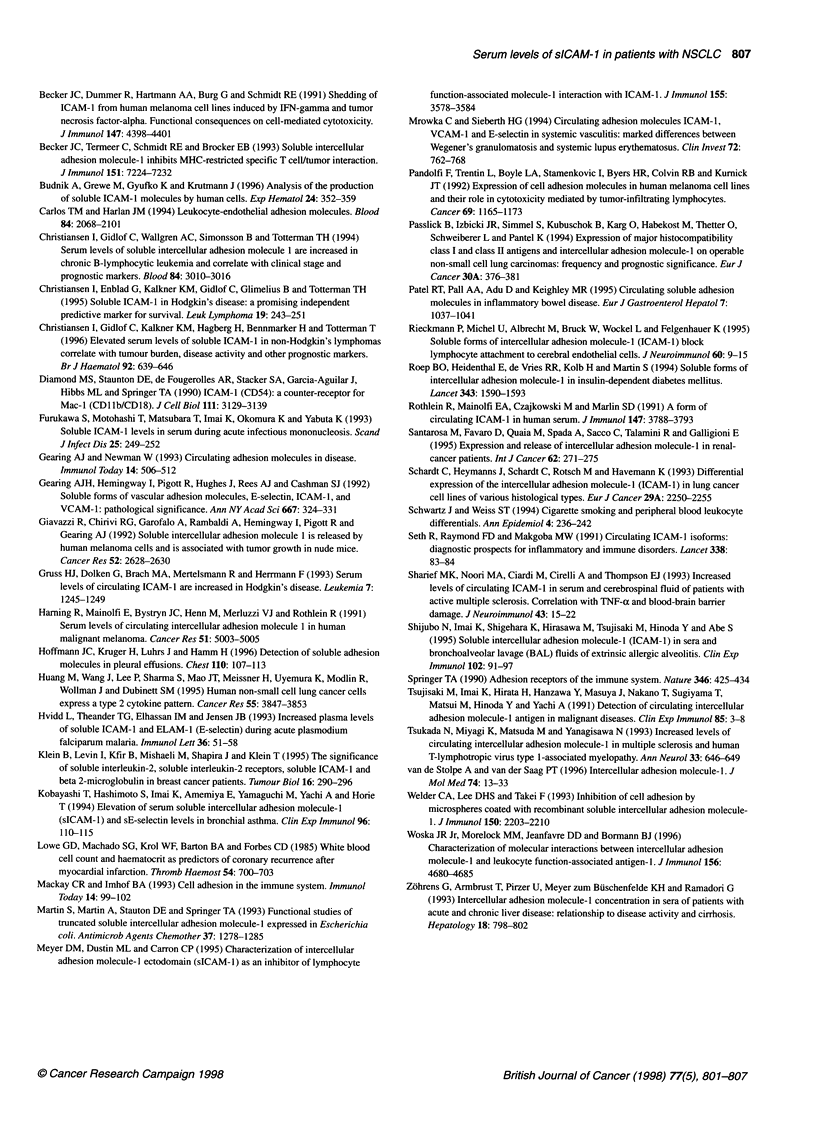# Serum levels of soluble intercellular adhesion molecule-1 (ICAM-1, CD54) in patients with non-small-cell lung cancer: correlation with histological expression of ICAM-1 and tumour stage.

**DOI:** 10.1038/bjc.1998.130

**Published:** 1998-03

**Authors:** A. Grothey, P. Heistermann, S. Philippou, R. Voigtmann

**Affiliations:** Department of Haematology, Marienhospital Herne, Ruhr-University of Bochum, Germany.

## Abstract

The expression of the intercellular adhesion molecule-1 (ICAM-1, CD54) seems to have an influence on the metastatic behaviour of tumour cells via immunological mechanisms. Recently, a soluble form of ICAM-1 was identified in physiological fluids. We analysed the serum levels of sICAM-1 in patients with non-small-cell lung cancer (NSCLC) and healthy individuals using a sandwich ELISA technique. Sera from 51 patients with NSCLC were tested for sICAM-1 (46 male, five female; age 38-81 years, median 64 years), 29 of whom presented with localized and 26 with metastatic disease. The control group consisted of 40 healthy individuals (20 smokers, 20 non-smokers). Immunohistochemical analysis of ICAM-1 in tumour cells was performed in 20 cases. Patients with NSCLC had significantly higher serum levels of sICAM-1 compared with healthy non-smokers (P = 0.00001) and smokers (P= 0.0328). Metastatic disease was associated with higher sICAM-1 than localized tumours (P = 0.0013). Only 11 out of 23 patients with localized NSCLC had sICAM-1 levels >300 ng ml(-1), compared with 25 out of 28 patients with metastatic disease. Histological expression of ICAM-1 was positively correlated with serum slCAM-1 (P = 0.0399). No difference was observed between histological tumour types with regard to sICAM-1 or NSCLC expression of ICAM-1. In sequential analysis (13 patients), rising sICAM-1 levels predicted a short-term fatal outcome (P = 0.0054) but, overall, sICAM-1 levels did not correlate with prognosis. In the control group, smokers showed significantly higher levels than non-smokers (P = 0.0016). In contrast to patients with NSCLC, sICAM-1 in the control group was correlated to the leucocyte count (r = 0.580, P = 0.003). In conclusion, serum levels of sICAM-1 seem to be associated with tumour burden and histological expression of ICAM-1 in patients with NSCLC. However, the (patho-) physiological role of ICAM-1 in NSCLC remains to be determined.


British Journal of Cancer (1998) 77(5), 801–807
© 1998 Cancer Research Campaign

### Serum levels of soluble intercellular adhesion molecule-1 (ICAM-1, CD54) in patients with non-small-cell lung cancer: correlation with histological expression of ICAM-1 and tumour stage

A Grothey[1]*, P Heistermann[2], S Philippou[3] and R Voigtmann[1]

Departments of [1]Haematology and Oncology and [2]Department of Surgery, Marienhospital Herne, Ruhr-University of Bochum, Hoelkeskampring 40, 44625 Herne, Germany; [3]Institute of Pathology, Ruhr-University of Bochum, Universitaetsstr. 150, 44780 Bochum, Germany

**Summary** The expression of the intercellular adhesion molecule-1 (ICAM-1, CD54) seems to have an influence on the metastatic behaviour of tumour cells via immunological mechanisms. Recently, a soluble form of ICAM-1 was identified in physiological fluids. We analysed the serum levels of sICAM-1 in patients with non-small-cell lung cancer (NSCLC) and healthy individuals using a sandwich ELISA technique. Sera from 51 patients with NSCLC were tested for sICAM-1 (46 male, five female; age 38–81 years, median 64 years), 29 of whom presented with localized and 26 with metastatic disease. The control group consisted of 40 healthy individuals (20 smokers, 20 non-smokers). Immunohistochemical analysis of ICAM-1 in tumour cells was performed in 20 cases. Patients with NSCLC had significantly higher serum levels of sICAM-1 compared with healthy non-smokers ($P$ = 0.00001) and smokers ($P$ = 0.0328). Metastatic disease was associated with higher sICAM-1 than localized tumours ($P$ = 0.0013). Only 11 out of 23 patients with localized NSCLC had sICAM-1 levels >300 ng ml$^{-1}$, compared with 25 out of 28 patients with metastatic disease. Histological expression of ICAM-1 was positively correlated with serum sICAM-1 ($P$ = 0.0399). No difference was observed between histological tumour types with regard to sICAM-1 or NSCLC expression of ICAM-1. In sequential analysis (13 patients), rising sICAM-1 levels predicted a short-term fatal outcome ($P$ = 0.0054) but, overall, sICAM-1 levels did not correlate with prognosis. In the control group, smokers showed significantly higher levels than non-smokers ($P$ = 0.0016). In contrast to patients with NSCLC, sICAM-1 in the control group was correlated to the leucocyte count ($r$ = 0.580, $P$ = 0.003). In conclusion, serum levels of sICAM-1 seem to be associated with tumour burden and histological expression of ICAM-1 in patients with NSCLC. However, the (patho-) physiological role of ICAM-1 in NSCLC remains to be determined.

**Keywords:** ICAM-1; soluble ICAM-1; lung cancer; smoking

Cellular adhesion molecules (CAMs) mediate a great variety of homotypic and heterotypic cellular interactions. By their molecular structures, CAMs can be divided into four major subgroups: cadherins, selectins, an immunoglobulin superfamily and integrins (Springer, 1990). In the context of human malignancy, the intercellular adhesion molecule-1 (ICAM-1, CD54) appears to be of special interest because it may be involved in the process of metastatic spread.

ICAM-1 belongs to the immunoglobulin gene superfamily and is the ligand for the β2-integrins, LFA-1 (leucocyte function-associated antigen-1, CD11a/CD18) and Mac-1 (CD11b/CD18) (Diamond et al, 1990; Carlos and Harlan, 1994). The expression of ICAM-1 on the cell surface can be induced by several cytokines (e.g. IFN-γ, TNF-α) and also by lipopolysaccharid (LPS), oxygen radicals and hypoxia. It is then present in white blood cells, fibroblasts, endothelial cells and some epithelial tissues (van de Stolpe and van der Saag, 1996).

The ICAM-1-mediated contact between leucocytes and endothelial cells is a mandatory step in the interaction of these two cell systems, i.e. during inflammatory processes. This phenomenon can be used as a model for the interaction between circulating cells and blood vessel walls (Adams and Shaw, 1994). Tumour cells imitating leucocytes in their surface adhesion molecules could use this pathway for vascular adhesion in the process of haematogenous metastasis (Aznavoorian et al, 1993).

Furthermore, ICAM-1 is an accessory molecule stabilizing the T-cell receptor-mediated binding between antigen-presenting cells (APC) and T lymphocytes. Thus, it serves as a cofactor in the activation of the cellular immune response (Springer, 1990; Mackay and Imhof, 1993). An immune reaction against a tumour would therefore more likely be directed against ICAM-1-expressing tumour cells (Pandolfi et al, 1992). On the other hand, release of soluble ICAM-1 molecules (sICAM-1) by the tumour could block the interaction between tumour cells, the APC and T lymphocytes causing peritumoral immunosuppression (Giavazzi et al, 1992). This immunosuppressive potential of sICAM-1 has been demonstrated in several in vitro models (Becker et al, 1991, 1993; Altomonte et al, 1993; Roep et al, 1994).

Elevated serum levels of sICAM-1 have been identified in various inflammatory, infectious and malignant diseases (Rothlein et al, 1991; Seth et al, 1991; Tsujisaki et al, 1991), including pancreatic carcinoma (Santarosa et al, 1995; Banks

*Received 23 January 1997*
*Revised 28 July 1997*
*Accepted 31 July 1997*

*Correspondence to:* A Grothey, Department of Biochemistry and Molecular Biology, Box 117, MD Anderson Cancer Center, 1515 Holcombe Blvd., Houston, TX 77030, USA

**Present address:* Department of Biochemistry and Molecular Biology, Box 117, MD Anderson Cancer Center, 1515 Holcombe Blvd., Houston, TX 77030, USA

et al, 1993; Gearing and Newman, 1993), malignant melanoma (Harning et al, 1991), breast cancer (Klein et al, 1995) and lymphomas (Gruss et al, 1993; Christiansen et al, 1996). They are associated with an unfavourable prognosis in chronic lymphatic leukaemia and Hodgkin's disease (Christiansen et al, 1994, 1995).

In non-small-cell lung cancer (NSCLC), ICAM-1 expression has been demonstrated histologically, in tumour cell lines and as a soluble molecule in supernatants of tumour cell cultures (Schardt et al, 1993; Passlick et al, 1994). As yet, serum levels of sICAM-1 have not been compared with tumour stage and tumour cell expression of ICAM-1 in NSCLC.

We therefore analysed the serum levels of sICAM-1 in patients with NSCLC and a control group of healthy smokers and non-smokers and correlated our findings with the histological expression of ICAM-1 in the respective tumours.

**Table 1** Distribution of patients, stage, resectability and histological tumour type for sICAM-1 analysis

| Stage/Resection | | Histology | | | |
|---|---|---|---|---|---|
| | *n* | Squamous | Adenocarcinoma | Large-cell | Mixed |
| Metastatic | 28 | 14 | 10 | 3 | 1 |
| Localized | 23 | 10 | 8 | 4 | 1 |
| R2/inoperable | 9 | 6 | 1 | 2 | 0 |
| R1/R0 | 9 | 0 | 7 | 1 | 1 |
| Functionally inoperable | 5 | 4 | 0 | 1 | 0 |
| Total | 51 | 24 | 18 | 7 | 2 |

**Table 2** Clinical course and sICAM-1 in 13 patients with sequential analysis of sICAM-1 serum levels

| Patients | Stage | S1 sICAM-1 (ng ml⁻¹) | S2 Tx after S1 (time interval S1–S2) | S2 sICAM-1 status | S2 Tx after S2 (time interval S2–S3) | S3 sICAM-1 status |
|---|---|---|---|---|---|---|
| 1 | Localized inoperable | 602.8 | RTx (6 months) | 225.6 PR | None (6 months) | 221.1 NC |
| 2 | Metastatic | 544.3 | CTx/RTx (3 months) | 654.6 PD | None (3 months) | 799.4 PD |
| 3 | Localized | 286.5 | R1 resection (6 months) | 225.3 NC | None (6 months) | 199.2 NC |
| 4 | Metastatic | 333.4 | CTx (6 months) | 313.7 NC | CTx (6 months) | 311.8 PD |
| 5 | Metastatic | 586.2 | CTx/RTx (3 months) | 700.0 PD | CTx (3 months) | 724.9 PD |
| 6 | Metastatic | 457.3 | CTx (3 months) | 338.2 MR | None (3 months) | 355.2 PD |
| 7 | Localized inoperable | 409.3 | RTx (6 months) | 290.0 PR | None (6 months) | 307.7 NC |
| 8 | Localized inoperable | 269.5 | CTx (6 months) | 234.8 NC | None (6 months) | 237.3 NC |
| 9 | Metastatic | 435.2 | CTx (3 months) | 370.5 NC | None (3 months) | 462.0 PD |
| 10 | Metastatic | 355.3 | CTx/RTx (3 months) | 357.3 NC | CTx (3 months) | 276.8 PD |
| 11 | Metastatic | 539.9 | CTx (3 months) | 503.1 PD | None (3 months) | 677.1 PD Pneumonia |
| 12 | Localized inoperable | 409.0 | CTx (3 months) | 429.7 NC | None (3 months) | 429.0 NC |
| 13 | Localized | 259.0 | R2 resection/RTx (6 months) | 272.7 NC | None (6 months) | 275.1 NC |

S1, S2, S3: time points of serum samples obtained in the course of the disease (S1, pretreatment). CTx, chemotherapy; RTx, radiotherapy; PR/MR, partial/minor remission; NC, no change; PD, progressive disease.

© Cancer Research Campaign 1998

### PATIENTS AND METHODS

Blood samples were obtained from patients with histologically confirmed NSCLC before surgical treatment, chemotherapy or radiation. Samples were centrifuged and the serum was stored at –20°C.

Sera from 51 patients with NSCLC were tested for sICAM-1 (46 male, five female; age 38–81 years, median 64 years), 23 of whom presented with localized (i.e. confined to the thorax) and 28 with metastatic disease. Only 7 of these 51 patients (13.7%; five male, two female) were non-smokers or had stopped smoking at least 5 years ago. Patients with non-metastatic disease (localized) were divided into three different subgroups according to the resectability of the tumours using R criteria (R0, no; R1, histological; R2, macroscopic residual tumour tissue). A tumour was classified as 'functionally inoperable' when non-tumour reasons (e.g. pulmonary or cardiovascular co-morbidity) prevented a surgical approach of a potentially resectable tumour. Staging of all patients was completed before the knowledge of sICAM-1 serum levels. The histological differentiation of the tumours showed 24 squamous cell, 18 adenocarcinomas, seven large-cell and two mixed-type carcinomas (Table 1). In 13 patients (all male, 6 adenocarcinoma, four squamous cell and three large-cell carcinomas), a sequential analysis of three serum samples (S1, S2, S3) obtained at different times in the course of the disease was performed retrospectively. In this assessment, all patients were included for whom at least three serum samples were available and who had survived at least 6 months after S1 (pretreatment). In view of great inter-individual differences in clinical course and overall survival, we tried to adjust the time intervals between S1–S2 and S2–S3 to 3 or 6 months depending on patient survival (<12 or >12 months after S1 respectively). Table 2 presents the individual time intervals and important clinical data in relation to the time points of serum collection.

The sICAM-1 control group consisted of 40 healthy individuals (20 male, 20 female; age 21–64 years, median 30.5 years) with 20 non-smokers and 20 smokers.

None of the patients with NSCLC showed signs of infection at the time of serum collection. All apparently healthy blood sample donors were free of disease symptoms, none took a continuous medication. In order to disclose hidden abnormalities, they underwent a laboratory test routine to obtain basic haematological parameters, including complete blood cell count, bilirubin, total serum protein, creatinin, alanine aminotransferase (ALT), calcium, potassium, sodium, C-reactive protein (CRP) and erythrocyte sedimentation rate (ESR). No abnormalities were found in any of the 40 sera of the healthy donors.

Serum levels of sICAM-1 were measured with a commercial ELISA kit (Diagnostic Products, R&D Systems, Minneapolis, MN, USA) using a sandwich ELISA technique following the manufacturer's instructions. In brief, 100 µl of serum samples diluted 1:20 with sample diluent and 100 µl of anti-ICAM-1 HRP-conjugated antibody were simultaneously added to a 96-well microtitre plate precoated with murine antibody against human ICAM-1. After 1.5 h incubation at room temperature, the wells were washed six times with 300 µl of factory-provided wash buffer. Immediately after the last washing, each well was filled with 100 µl of HRP substrate (tetramethylbenzidine) and incubated for 30 min at room temperature. The substrate reaction was stopped by adding 100 µl of provided acid solution per well. Optical density (OD) was measured at 450 nm with a correction wavelength of 620 nm using a microtitre plate reader. All assays were performed with duplicates of serum samples. The concentration of sICAM-1 of each unknown sample was calculated using the mean OD of the duplicates corresponding to the absorbance of a standard curve obtained from factory-provided standards. The test assay showed the following performance characteristics: sensitivity, 0.35 ng ml$^{-1}$; intra-assay coefficient of variation (CV), 4.4%; interassay, 6.5%. Storage stability of sICAM-1 at –20°C and no significant loss of concentration between 0 and 5 freeze–thaw cycles had been demonstrated.

ICAM-1 expression in NSCLC specimens was analysed using an immunohistochemical approach. Twenty tumour samples were available for analysis (ten adenocarcinomas, seven squamous cell and three large-cell carcinomas), of which eight had been obtained by surgical tumour resection and 12 by bronchoscopy or fine-needle biopsy. The immunohistological analysis was performed with a mouse monoclonal antibody followed by a biotin–streptavidin-based detection procedure. Sections of paraffin-embedded tissue samples were incubated at 37°C overnight, then deparaffined

**Table 3** Serum levels of sICAM-1 in patients with NSCLC and the control group

| | | sICAM-1 (ng ml$^{-1}$) | |
|---|---|---|---|
| **Test group** | ***n*** | **Median** | **95% Confidence interval** |
| Metastatic NSCLC | 28 | 404.0 | 387.3–540.3 |
| Localized NSCLC | 23 | 280.6 | 278.8–356.7 |
| R2/inoperable | 9 | 360.3 | 294.0–448.0 |
| R1/R0 | 9 | 261.3 | 248.7–321.0 |
| Functionally inoperable | 5 | 259.0 | 215.8–346.9 |
| Control group | 40 | 253.8 | 243.4–288.7 |
| Smokers | 20 | 312.0 | 269.3–341.1 |
| Non-smokers | 20 | 225.2 | 212.9–240.9 |

**Figure 1** Distribution and median of sICAM-1 in subgroups of patients with NSCLC and in the control group. Dashed line denotes upper normal serum level of sICAM-1. ○, Control group; ▲, localized NSCLC; ■, metastatic NSCLC

© Cancer Research Campaign 1998

and rehydrated. After washing with phosphate-buffered saline (PBS) for 10 min, 200 µl of 0.03% hydrogen peroxide was added for 30 min to block endogenous peroxidase. Afterwards, slides were incubated with 200 µl of preimmune goat serum for 20 min to minimize unspecific reactions. Then, 200 µl of mouse anti-human ICAM-1 (Boehringer Ingelheim Bioproducts, Heidelberg, Germany) was added at 1:20 dilution in PBS and incubated at room temperature for 1 h. After two washes with PBS (each 10 min) samples were coated with 200 µl of 1:200 diluted biotinylated goat anti-mouse antibody (Sigma Chemical, St Louis, MO, USA) followed by a 30-min incubation with Vectastain ABC reagent (Vector Laboratories, Burlingame, CA, USA) at room temperature. Then, three washes with PBS were performed before 500 µl of 3-amino-9-ethylcarbazole (AEC) solution (Sigma) was applied for 20 min at 37°C. After another wash with PBS, slides were briefly stained with haematoxylin and finally washed with water. Embedding was performed in glycerol. Representative areas of tumour samples were used for microscopic analysis. The relative percentage of ICAM-1-positive tumour cells (red stain) was determined as a semiquantitative marker for ICAM-1 expression. For comparative analysis, tumours were grouped into four categories (0–1, 1–30, 30–60, >60% positive cells).

Statistical calculations were performed with StatView 4.51 (Abacus Concepts, Berkeley, CA, USA). As not all results followed a normal distribution, the non-parametric Mann–Whitney test was used for data analysis, unless otherwise stated.

### RESULTS

The distribution of patients with tumour stage, surgical performance and histological tumour type is shown in Table 1.

The distribution of histological types was as expected for NSCLC, with one striking feature – no squamous cell carcinoma underwent a R1 or R0 resection in contrast to seven of the eight localized adenocarcinomas.

Serum levels of sICAM-1 showed great differences between patients with metastatic and localized disease and the controls. Table 3 presents the median sICAM-1 values and 95% confidence intervals of the subgroups. The distribution of individual test results according to tumour stage and resectability is given in Figure 1.

No age dependency of serum sICAM-1 was observed, neither in patients with NSCLC nor in the control group (Spearman correlation $P$ = 0.1942 and $P$ = 0.2352 respectively). Patients with metastatic disease showed significantly higher sICAM-1 levels compared with all other groups, including localized tumours ($P$ = 0.0013), except for the subgroup of patients with R2/inoperable tumours ($P$ = 0.2427). Only 11 of 23 patients with localized NSCLC presented with sICAM-1 levels >300 ng ml$^{-1}$ compared with 25 of 28 patients with metastatic disease. Localized tumours were associated with significantly elevated serum levels of sICAM-1 compared with the control group as a whole ($P$ = 0.0189). Among the different subgroups of non-metastatic NSCLC, there was a trend towards higher sICAM-1 levels in patients with R2/inoperable tumours (vs R1/R0, $P$ = 0.0703). In patients with metastatic disease, no association could be observed between sICAM-1 and the number, size or localization of metastases or involvement of specific organs. Serum levels of sICAM-1 seemed to be independent of the histological tumour type, no significant differences could be disclosed, with only a trend towards higher levels in squamous cell carcinomas (data not shown).

**Figure 2** sICAM-1 in the course of the disease in 13 patients with NSCLC. S1–S3, serum samples obtained at different times. †Patients not alive 3 months after S3. *Patients alive 3 months after S3. †vs *, S1 $P$ = 0.1877, S3 $P$ = 0.0054 ($U$-test). Values in brackets represent months of survival after S3. For detailed description of the clinical course in patient 1 and patient 2, see text (also compare with Table 2)

**Figure 3** Serum levels and median of sICAM-1 in patients with NSCLC in correlation with histological expression of ICAM-1, stated as percentage of ICAM-1-positive tumour cells. Dashed line denotes upper normal level of sICAM-1 in serum

Figure 2 presents sICAM-1 levels of 13 patients in consecutive serum samples, obtained at three different times (S1, S2, S3) in the course of their disease, and months of survival after S3. Whereas sICAM-1 levels at S1 did not differ significantly between patients who had or had not survived 3 months after S3 ($P$ = 0.1877), levels at S3 could almost exactly distinguish between these two groups ($P$ = 0.0054). Only one patient with sICAM-1 below 300 ng ml$^{-1}$ did not survive the following 3 months, and only one patient showing a level slightly above this limit (i.e. 307.7 ng ml$^{-1}$) was alive after this time.

Two very divergent patient histories might further illustrate the relation between sICAM-1 serum levels and clinical features in NSCLC (also see Figure 2 and Table 2). Patient 1 (male, age

© Cancer Research Campaign 1998

**Figure 4** White blood cell count and sICAM-1 serum levels in the control group. ○, Smokers; ●, non-smokers

60 years) presented with an inoperable adenocarcinoma of the left lower lobe ($T_3N_2M_0$) and a sICAM-1 serum level of 602.8 ng ml$^{-1}$. Radiotherapy of the primary tumour and lymph nodes achieved good clinical remission. Serum levels of sICAM-1 decreased to 225.6 ng ml$^{-1}$ 6 months after diagnosis (3 months after end of radiotherapy) and remained at that level after another 6 months (221.1 ng ml$^{-1}$). The patient is presently alive 15 months after S3.

In patient 2 (male, age 68 years), an hilar adenocarcinoma was diagnosed with bone and liver metastases ($T_4N_3M_1$). Serum sICAM-1 at time of diagnosis was 544.3 ng ml$^{-1}$. Chemotherapy and radiotherapy of the left hilar region were initiated with stable disease for 3 months, but eventually the tumour progressed in all locations. At that time the serum level of sICAM-1 was 654.6 ng ml$^{-1}$. The clinical status subsequently showed a rapid deterioration and sICAM-1 rose to 799.4 ng ml$^{-1}$ after another 3 months. The patient died 1 month later.

Table 2 outlines the clinical course of all 13 patients in relation to sICAM-1 at S1, S2 and S3. Overall, serum levels of sICAM-1 seemed to parallel the clinical course of patients with NSCLC.

Tumour expression of ICAM-1, assessed by immunohistochemistry in 20 NSCLCs, was independent of the histological tumour type (chi-square test $P$ = 0.3819, data not shown). A comparison between the immunohistological results and respective serum levels of soluble ICAM-1 revealed a significant, positive correlation (Kruskal–Wallis test $P$ = 0.0399). Only one of five patients with tumours displaying 0–1% ICAM-1-positive cells had serum levels > 300 ng ml$^{-1}$, compared with seven of eight and five of six patients with tumours showing 1–30% and 30–60% positivity respectively. Figure 3 presents the distribution of serum levels and histological expression of (s)ICAM-1.

We were able to obtain follow-up data in 32 of 51 patients (63%). When divided at the median of serum sICAM-1 (400 ng ml$^{-1}$), patients with higher sICAM-1 levels had a median survival of 4.5 months compared with 6 months in patients with sICAM-1 < 400 ng ml$^{-1}$ (log-rank $P$ = 0.5974). A cut-off at the upper normal limit of sICAM-1 levels (300 ng ml$^{-1}$) was also not able to distinguish between groups of patients with difference in prognosis (log rank $P$ = 0.6782). Thus, sICAM-1 obtained at time of diagnosis was not a prognostic factor concerning overall survival. The histological expression of ICAM-1 also appeared to have no influence on survival (log-rank $P$ = 0.6306).

In the healthy control group, a striking difference was observed between the sICAM-1 levels of smokers and non-smokers, with significantly higher levels in smokers ($P$ = 0.0016), which did not differ from patients with localized NSCLC ($P$ = 0.8838). In order to explain the unexpectedly high sICAM-1 levels in smokers, we tried to correlate the individual sICAM-1 result with other characteristics, such as numbers of cigarettes smoked per day and leucocyte counts. No association between quantity of cigarette consumption and sICAM-1 could be disclosed, but there was a trend towards a correlation between leucocyte count and sICAM-1 (Spearman's rank correlation $r$ = 0.417, $P$ = 0.0687), which became significant when non-smokers were included ($r$ = 0.580, $P$ = 0.003; Figure 4). Therefore, in the control group, elevated sICAM-1 levels seemed to be closely related to leucocytosis. In contrast to this, no correlation between sICAM-1 levels and individual leucocyte counts could be found in patients with NSCLC ($r$ = 0.153, $P$ = 0.2822; data not shown), regardless of smoking behaviour ($P$ = 0.2854 for smokers and $P$ = 0.7264 for non-smokers). It is noteworthy that only 1 of 24 patients with squamous cell carcinomas, but 5 of 18 patients with adenocarcinomas, were non-smokers (chi-square test $P$ = 0.0274).

### DISCUSSION

We have shown that serum sICAM-1 levels were closely related to tumour stage in patients with NSCLC, with a remarkable difference between patients with metastatic and localized disease. Subgroups of the latter also showed a trend towards higher sICAM-1 values in more advanced tumours when subdivided according to the results of surgical performance and resectability.

In the 13 patients with sequential analysis of three serum samples in the course of the disease, a trend towards higher sICAM-1 level predicted a fatal outcome in the near future with statistical significance. However, in the 32 patients for whom survival data could be obtained, pretreatment sICAM-1 levels were not correlated with prognosis. Therefore, not the absolute sICAM-1 level, but its trend during tumour treatment, might be of relevance. Further investigations with sequential analysis of sICAM-1 levels are necessary to evaluate sICAM-1 as a marker for monitoring disease activity in patients with NSCLC.

Unexpectedly, serum levels in the total control group showed a great variation with a high number of values above the previously reported upper normal limit of 300 ng ml$^{-1}$ in ELISA (Banks et al, 1993). An analysis of subgroups revealed that healthy smokers presented with significantly higher sICAM-1 levels than non-smokers. This was probably because of the association of sICAM-1 and leucocyte counts demonstrated in the control group. We suppose that the well-known 'smokers' leucocytosis' (Lowe et al, 1985; Schwartz and Weiss, 1994) may account for the elevated serum levels in smokers that lay in the same range as those of patients with localized NSCLC. This possibility should be more carefully considered in future studies, as smokers could be regarded as a more obvious control group for patients with lung cancer than healthy non-smokers. However, in patients with NSCLC, no association between leucocyte counts and sICAM-1 could be disclosed, so that additional, unknown factors appear to have contributed to the elevation of sICAM-1, particularly in patients with metastatic disease.

© Cancer Research Campaign 1998

Elevated sICAM-1 levels have been demonstrated in several other malignant diseases (van de Stolpe and van der Saag, 1996), and also in non-malignant acute (infectious) and chronic diseases, such as acute infectious mononucleosis (Furukawa et al, 1993), *P. falciparum* malaria (Hvidd et al, 1993), autoimmune and viral hepatitis (Zöhrens et al, 1993), alcoholic liver cirrhosis (Zöhrens et al, 1993), extrinsic allergic alveolitis (Shijubo et al, 1995), acute attacks of bronchial asthma (Kobayashi et al, 1994), inflammatory bowel disease (Patel et al, 1995), systemic lupus erythematosus (Mrowka and Sieberth, 1994), active multiple sclerosis (Sharief et al, 1993; Tsukada et al, 1993; Rieckmann et al, 1995), diabetes (Roep et al, 1994) and chronic renal failure (Gearing et al, 1992).

The manner by which the soluble form of sICAM-1 is generated is presently under discussion. The molecule might represent the extracellular part of the membrane-bound ICAM-1 split off by proteolytic cleavage and shed into the extracellular fluid (van de Stolpe and van der Saag, 1996). Alternatively, sICAM-1 could be produced as a secreted splice variant of ICAM-1 lacking the intramembrane and intracellular domains, however no corresponding spliced mRNA has yet been identified (Budnik et al, 1996). Several circulating forms differing in molecular weight have been observed, suggesting that molecules might assemble to complexes and/or experience a cell type-dependent glycosylation (Rothlein et al, 1991; Seth et al, 1991). It is noteworthy that different forms of sICAM-1, for example monomeric or complex and immobilized or circulating, have been associated with different functional characteristics of the molecules (Martin et al, 1993; Welder et al, 1993; Woska et al, 1996).

Although there are in vitro observations that tumour cells, including NSCLC, can express ICAM-1 and shed the soluble form (Schardt et al, 1993; Passlick et al, 1994) the source of sICAM-1 in vivo in patients with malignant diseases is uncertain. The main question closely linked to the functional importance and (patho)physiological role of sICAM-1 in cancer is: do the elevated serum levels reflect the production of sICAM-1 by tumour cells or is it just a non-specific phenomenon caused by inflammatory/immunological host reactions? That is, is sICAM-1 just another tumour marker with very low specificity or does it induce functional changes in the tumour–host interaction?

We observed a significant correlation between the serum levels of soluble sICAM-1 and the histological tumour expression of ICAM-1, suggesting that tumour cells are the source of serum sICAM-1. However, this correlation cannot serve as firm evidence that sICAM-1 released by tumour cells solely contributes to the elevated serum levels found in patients with NSCLC. In this regard, two caveats have to be considered. Firstly, the fact that an increase of sICAM-1 has been demonstrated in a great variety of benign and malignant, acute and chronic diseases suggests that elevated sICAM-1 levels at least partly represent a reactive phenomenon that is likely due to non-specific host defence mechanisms. This is further supported by our finding of elevated serum levels in healthy smokers. In addition, a recent analysis of soluble ICAM-1 in pleural effusions showed no difference between inflammatory and malignant exsudates (Hoffmann et al, 1996). Secondly, in 12 of our 20 cases, only small tumour samples obtained by bronchoscopy or fine-needle biopsy were available for immunohistological analysis. Considering tumour heterogeneity, the examined samples might not be representative of the predominant differentation of the tumour in these cases. This might also explain why, in contrast to previous histological findings and observations in cell cultures, whereas an ICAM-1 expression was most prevalent in squamous cell carcinomas (Schardt et al, 1993), we found no difference in sICAM-1 serum levels and histological ICAM-1 expression between the various histological tumour types.

As membrane-bound ICAM-1 serves as a co-stimulatory factor for the T-cell receptor-mediated cellular immune response, a shedding and loss of ICAM-1 in tumour cells might represent one mechanism of immune escape (Springer, 1990). Additionally, sICAM-1 could block the T-cell–APC interaction or at least modify the integrin-mediated functional status of leucocytes (Giavazzi et al, 1992). In this way, sICAM-1 could act as an immunosuppressive agent. In patients with diabetes mellitus, the immunosuppressive capacity of elevated serum levels of sICAM-1 has been demonstrated in vitro (Roep et al, 1994), although the relevance of these findings for the in vivo situation has recently been questioned (Meyer et al, 1995). In malignant tumours, one has to keep in mind that the observed systemic levels might not necessarily reflect the local, peritumoral increases that could create a localized immunosuppression, with a survival advantage for tumour cell shedding sICAM-1. This possible tumour defence mechanism against the host immune system is similar to other observations, such as the frequent loss of HLA antigens on tumour cells or the $T_{H2}$ pattern of cytokine release recently found in NSCLC (Huang et al, 1995). Clarifying the role of ICAM-1 in the interaction tumour–immune system could have several implications for the generation of tumour vaccines in the treatment of cancer.

Our study clearly demonstrates the association of advanced, metastatic tumour stages in NSCLC with an elevation of sICAM-1 in the sera of patients. The correlation of sICAM-1 serum levels and tumour expression of ICAM-1 suggests that a release of soluble ICAM-1 by tumour cells at least contributes to this phenomenon. We did not address the question of potential consequences of elevated sICAM-1 for the immune status. Future investigations should try to further establish the connection between the histological expression of ICAM-1 in the original tumour, the in vitro characteristics of individual tumour cell cultures, including the shedding of sICAM-1, and the in vivo findings in patients with regard to sICAM-1 serum levels, clinical tumour stage, prognosis and immune status.

Finally, a sidenote resulting from our study deals with the question of defining 'normal values' for sICAM-1 in serum. The obvious correlation between leucocytosis/smoking habit and sICAM-1 in the healthy control group with an increase of up to twofold in smokers compared with non-smokers might have important implications for other investigations analysing sICAM-1 levels in a variety of pathological circumstances. As with other serum proteins, e.g. CEA, an individual test result for sICAM-1 has to be critically evaluated in view of other individual factors, such as leucocytosis and smoking habits.

### REFERENCES

Adams DH and Shaw S (1994) Leucocyte–endothelial interactions and regulation of leucocyte migration. *Lancet* **343**: 831–836

Altomonte M, Gloghini A, Bertola G, Gasparollo A, Carbone A, Ferrone S and Maio M (1993) Differential expression of cell adhesion molecules CD54/CD11a and CD58/CD2 by human melanoma cells and functional role in their interaction with cytotoxic cells. *Cancer Res* **53**: 3343–3348

Aznavoorian S, Murphy AN, Stetler-Stevenson WG and Liotta LA (1993) Molecular aspects of tumor cell invasion and metastasis. *Cancer* **71**: 1368–1383

Banks RE, Gearing AJ, Hemingway IK, Norfolk DR, Perren TJ and Selby PJ (1993) Circulating intercellular adhesion molecule-1 (ICAM-1), E-selectin and vascular cell adhesion molecule-1 (VCAM-1) in human malignancies. *Br J Cancer* **68**: 122–124

© Cancer Research Campaign 1998

Becker JC, Dummer R, Hartmann AA, Burg G and Schmidt RE (1991) Shedding of ICAM-1 from human melanoma cell lines induced by IFN-gamma and tumor necrosis factor-alpha. Functional consequences on cell-mediated cytotoxicity. *J Immunol* **147**: 4398–4401

Becker JC, Termeer C, Schmidt RE and Brocker EB (1993) Soluble intercellular adhesion molecule-1 inhibits MHC-restricted specific T cell/tumor interaction. *J Immunol* **151**: 7224–7232

Budnik A, Grewe M, Gyufko K and Krutmann J (1996) Analysis of the production of soluble ICAM-1 molecules by human cells. *Exp Hematol* **24**: 352–359

Carlos TM and Harlan JM (1994) Leukocyte-endothelial adhesion molecules. *Blood* **84**: 2068–2101

Christiansen I, Gidlof C, Wallgren AC, Simonsson B and Totterman TH (1994) Serum levels of soluble intercellular adhesion molecule 1 are increased in chronic B-lymphocytic leukemia and correlate with clinical stage and prognostic markers. *Blood* **84**: 3010–3016

Christiansen I, Enblad G, Kalkner KM, Gidlof C, Glimelius B and Totterman TH (1995) Soluble ICAM-1 in Hodgkin's disease: a promising independent predictive marker for survival. *Leuk Lymphoma* **19**: 243–251

Christiansen I, Gidlof C, Kalkner KM, Hagberg H, Bennmarker H and Totterman T (1996) Elevated serum levels of soluble ICAM-1 in non-Hodgkin's lymphomas correlate with tumour burden, disease activity and other prognostic markers. *Br J Haematol* **92**: 639–646

Diamond MS, Staunton DE, de Fougerolles AR, Stacker SA, Garcia-Aguilar J, Hibbs ML and Springer TA (1990) ICAM-1 (CD54): a counter-receptor for Mac-1 (CD11b/CD18). *J Cell Biol* **111**: 3129–3139

Furukawa S, Motohashi T, Matsubara T, Imai K, Okomura K and Yabuta K (1993) Soluble ICAM-1 levels in serum during acute infectious mononucleosis. *Scand J Infect Dis* **25**: 249–252

Gearing AJ and Newman W (1993) Circulating adhesion molecules in disease. *Immunol Today* **14**: 506–512

Gearing AJH, Hemingway I, Pigott R, Hughes J, Rees AJ and Cashman SJ (1992) Soluble forms of vascular adhesion molecules, E-selectin, ICAM-1, and VCAM-1: pathological significance. *Ann NY Acad Sci* **667**: 324–331

Giavazzi R, Chirivi RG, Garofalo A, Rambaldi A, Hemingway I, Pigott R and Gearing AJ (1992) Soluble intercellular adhesion molecule 1 is released by human melanoma cells and is associated with tumor growth in nude mice. *Cancer Res* **52**: 2628–2630

Gruss HJ, Dolken G, Brach MA, Mertelsmann R and Herrmann F (1993) Serum levels of circulating ICAM-1 are increased in Hodgkin's disease. *Leukemia* **7**: 1245–1249

Harning R, Mainolfi E, Bystryn JC, Henn M, Merluzzi VJ and Rothlein R (1991) Serum levels of circulating intercellular adhesion molecule 1 in human malignant melanoma. *Cancer Res* **51**: 5003–5005

Hoffmann JC, Kruger H, Luhrs J and Hamm H (1996) Detection of soluble adhesion molecules in pleural effusions. *Chest* **110**: 107–113

Huang M, Wang J, Lee P, Sharma S, Mao JT, Meissner H, Uyemura K, Modlin R, Wollman J and Dubinett SM (1995) Human non-small cell lung cancer cells express a type 2 cytokine pattern. *Cancer Res* **55**: 3847–3853

Hvidd L, Theander TG, Elhassan IM and Jensen JB (1993) Increased plasma levels of soluble ICAM-1 and ELAM-1 (E-selectin) during acute plasmodium falciparum malaria. *Immunol Lett* **36**: 51–58

Klein B, Levin I, Kfir B, Mishaeli M, Shapira J and Klein T (1995) The significance of soluble interleukin-2, soluble interleukin-2 receptors, soluble ICAM-1 and beta 2-microglobulin in breast cancer patients. *Tumour Biol* **16**: 290–296

Kobayashi T, Hashimoto S, Imai K, Amemiya E, Yamaguchi M, Yachi A and Horie T (1994) Elevation of serum soluble intercellular adhesion molecule-1 (sICAM-1) and sE-selectin levels in bronchial asthma. *Clin Exp Immunol* **96**: 110–115

Lowe GD, Machado SG, Krol WF, Barton BA and Forbes CD (1985) White blood cell count and haematocrit as predictors of coronary recurrence after myocardial infarction. *Thromb Haemost* **54**: 700–703

Mackay CR and Imhof BA (1993) Cell adhesion in the immune system. *Immunol Today* **14**: 99–102

Martin S, Martin A, Stauton DE and Springer TA (1993) Functional studies of truncated soluble intercellular adhesion molecule-1 expressed in *Escherichia coli. Antimicrob Agents Chemother* **37**: 1278–1285

Meyer DM, Dustin ML and Carron CP (1995) Characterization of intercellular adhesion molecule-1 ectodomain (sICAM-1) as an inhibitor of lymphocyte function-associated molecule-1 interaction with ICAM-1. *J Immunol* **155**: 3578–3584

Mrowka C and Sieberth HG (1994) Circulating adhesion molecules ICAM-1, VCAM-1 and E-selectin in systemic vasculitis: marked differences between Wegener's granulomatosis and systemic lupus erythematosus. *Clin Invest* **72**: 762–768

Pandolfi F, Trentin L, Boyle LA, Stamenkovic I, Byers HR, Colvin RB and Kurnick JT (1992) Expression of cell adhesion molecules in human melanoma cell lines and their role in cytotoxicity mediated by tumor-infiltrating lymphocytes. *Cancer* **69**: 1165–1173

Passlick B, Izbicki JR, Simmel S, Kubuschok B, Karg O, Habekost M, Thetter O, Schweiberer L and Pantel K (1994) Expression of major histocompatibility class I and class II antigens and intercellular adhesion molecule-1 on operable non-small cell lung carcinomas: frequency and prognostic significance. *Eur J Cancer* **30A**: 376–381

Patel RT, Pall AA, Adu D and Keighley MR (1995) Circulating soluble adhesion molecules in inflammatory bowel disease. *Eur J Gastroenterol Hepatol* **7**: 1037–1041

Rieckmann P, Michel U, Albrecht M, Bruck W, Wockel L and Felgenhauer K (1995) Soluble forms of intercellular adhesion molecule-1 (ICAM-1) block lymphocyte attachment to cerebral endothelial cells. *J Neuroimmunol* **60**: 9–15

Roep BO, Heidenthal E, de Vries RR, Kolb H and Martin S (1994) Soluble forms of intercellular adhesion molecule-1 in insulin-dependent diabetes mellitus. *Lancet* **343**: 1590–1593

Rothlein R, Mainolfi EA, Czajkowski M and Marlin SD (1991) A form of circulating ICAM-1 in human serum. *J Immunol* **147**: 3788–3793

Santarosa M, Favaro D, Quaia M, Spada A, Sacco C, Talamini R and Galligioni E (1995) Expression and release of intercellular adhesion molecule-1 in renal-cancer patients. *Int J Cancer* **62**: 271–275

Schardt C, Heymanns J, Schardt C, Rotsch M and Havemann K (1993) Differential expression of the intercellular adhesion molecule-1 (ICAM-1) in lung cancer cell lines of various histological types. *Eur J Cancer* **29A**: 2250–2255

Schwartz J and Weiss ST (1994) Cigarette smoking and peripheral blood leukocyte differentials. *Ann Epidemiol* **4**: 236–242

Seth R, Raymond FD and Makgoba MW (1991) Circulating ICAM-1 isoforms: diagnostic prospects for inflammatory and immune disorders. *Lancet* **338**: 83–84

Sharief MK, Noori MA, Ciardi M, Cirelli A and Thompson EJ (1993) Increased levels of circulating ICAM-1 in serum and cerebrospinal fluid of patients with active multiple sclerosis. Correlation with TNF-α and blood-brain barrier damage. *J Neuroimmunol* **43**: 15–22

Shijubo N, Imai K, Shigehara K, Hirasawa M, Tsujisaki M, Hinoda Y and Abe S (1995) Soluble intercellular adhesion molecule-1 (ICAM-1) in sera and bronchoalveolar lavage (BAL) fluids of extrinsic allergic alveolitis. *Clin Exp Immunol* **102**: 91–97

Springer TA (1990) Adhesion receptors of the immune system. *Nature* **346**: 425–434

Tsujisaki M, Imai K, Hirata H, Hanzawa Y, Masuya J, Nakano T, Sugiyama T, Matsui M, Hinoda Y and Yachi A (1991) Detection of circulating intercellular adhesion molecule-1 antigen in malignant diseases. *Clin Exp Immunol* **85**: 3–8

Tsukada N, Miyagi K, Matsuda M and Yanagisawa N (1993) Increased levels of circulating intercellular adhesion molecule-1 in multiple sclerosis and human T-lymphotropic virus type 1-associated myelopathy. *Ann Neurol* **33**: 646–649

van de Stolpe A and van der Saag PT (1996) Intercellular adhesion molecule-1. *J Mol Med* **74**: 13–33

Welder CA, Lee DHS and Takei F (1993) Inhibition of cell adhesion by microspheres coated with recombinant soluble intercellular adhesion molecule-1. *J Immunol* **150**: 2203–2210

Woska JR Jr, Morelock MM, Jeanfavre DD and Bormann BJ (1996) Characterization of molecular interactions between intercellular adhesion molecule-1 and leukocyte function-associated antigen-1. *J Immunol* **156**: 4680–4685

Zöhrens G, Armbrust T, Pirzer U, Meyer zum Büschenfelde KH and Ramadori G (1993) Intercellular adhesion molecule-1 concentration in sera of patients with acute and chronic liver disease: relationship to disease activity and cirrhosis. *Hepatology* **18**: 798–802

© Cancer Research Campaign 1998